# 1-{2-(4-Chloro­benz­yloxy)-2-[4-(mor­pho­lin-4-yl)phen­yl]eth­yl}-1*H*-benzimidazole propan-2-ol monosolvate

**DOI:** 10.1107/S1600536813022599

**Published:** 2013-08-17

**Authors:** Özden Özel Güven, Seval Çapanlar, Philip D. F. Adler, Simon J. Coles, Tuncer Hökelek

**Affiliations:** a Department of Chemistry, Bülent Ecevit University, 67100 Zonguldak, Turkey; b Department of Chemistry, Southampton University, SO17 1BJ Southampton, England; c Department of Physics, Hacettepe University, 06800 Beytepe, Ankara, Turkey

## Abstract

In the title compound, C_26_H_26_ClN_3_O_2_·C_3_H_7_OH, the benzimid­azole ring system is essentially planar [maximum deviation = −0.018 (2) Å] and its mean plane is oriented with respect to the two benzene rings at dihedral angles of 4.51 (6) and 56.16 (6)°, and the dihedral angle between the two benzene rings is 59.11 (7)°. The morpholine ring displays a chair conformation. The propan-2-ol solvent mol­ecule links with the benzimidazole ring *via* an O—H⋯N hydrogen bond. In the crystal, weak inter­molecular C—H⋯O hydrogen bonds link the mol­ecules into inversion dimers with an *R*_2_^2^(28) motif. π–π stacking occurs between the parallel chloro­benzene rings [centroid–centroid distance = 3.792 (1) Å]. Weak C—H⋯π inter­actions and short Cl⋯Cl [3.2037 (10) Å] contacts are also observed.

## Related literature

For general background to the biological activity of benz­imid­azole derivatives, see: Özel Güven *et al.* (2007*a* ▶,*b* ▶). For related structures, see: Caira *et al.* (2004 ▶); Freer *et al.* (1986 ▶); Özel Güven *et al.* (2008*a* ▶,*b* ▶,*c* ▶,*d* ▶, 2013 ▶); Peeters *et al.* (1979*a* ▶,*b* ▶, 1996 ▶). For ring puckering parameters, see: Cremer & Pople (1975 ▶). For ring motif details, see: Bernstein *et al.* (1995 ▶).
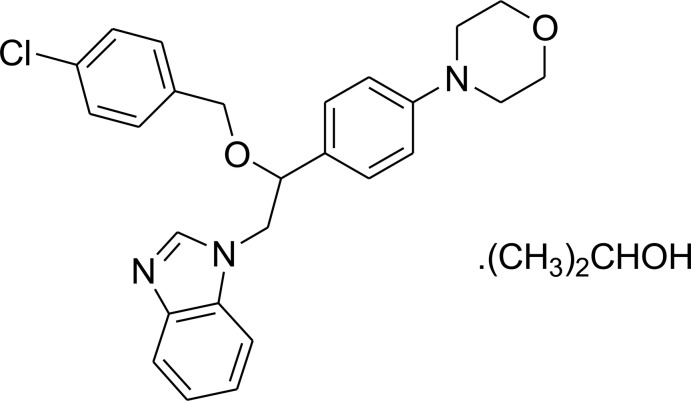


## Experimental

### 

#### Crystal data

C_26_H_26_ClN_3_O_2_·C_3_H_8_O
*M* *_r_* = 508.04
Triclinic, 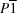

*a* = 10.6542 (3) Å
*b* = 11.5152 (4) Å
*c* = 11.6853 (4) Å
α = 87.010 (3)°
β = 83.703 (3)°
γ = 71.572 (2)°
*V* = 1351.66 (8) Å^3^
*Z* = 2
Mo *K*α radiation
μ = 0.18 mm^−1^
*T* = 294 K
0.30 × 0.28 × 0.25 mm

#### Data collection

Rigaku R-AXIS RAPID-S diffractometer
Absorption correction: multi-scan (*CrystalClear-SM Expert*; Rigaku, 2011 ▶) *T*_min_ = 0.95, *T*_max_ = 0.96
17368 measured reflections
6139 independent reflections
3561 reflections with *I* > 2σ(*I*)
*R*_int_ = 0.048

#### Refinement

*R*[*F* ^2^ > 2σ(*F* ^2^)] = 0.055
*wR*(*F* ^2^) = 0.186
*S* = 1.11
6139 reflections
332 parameters
1 restraint
H atoms treated by a mixture of independent and constrained refinement
Δρ_max_ = 0.50 e Å^−3^
Δρ_min_ = −0.53 e Å^−3^

### 

Data collection: *CrystalClear-SM Expert* (Rigaku, 2011 ▶); cell refinement: *CrystalClear-SM Expert*; data reduction: *CrystalClear-SM Expert*; program(s) used to solve structure: *SHELXS97* (Sheldrick, 2008 ▶); program(s) used to refine structure: *SHELXL97* (Sheldrick, 2008 ▶); molecular graphics: *ORTEP-3 for Windows* (Farrugia, 2012 ▶); software used to prepare material for publication: *WinGX* (Farrugia, 2012 ▶) and *PLATON* (Spek, 2009 ▶).

## Supplementary Material

Crystal structure: contains datablock(s) I, global. DOI: 10.1107/S1600536813022599/xu5728sup1.cif

Structure factors: contains datablock(s) I. DOI: 10.1107/S1600536813022599/xu5728Isup2.hkl

Supplementary material file. DOI: 10.1107/S1600536813022599/xu5728Isup3.cml

Additional supplementary materials:  crystallographic information; 3D view; checkCIF report

## Figures and Tables

**Table 1:** Hydrogen-bond geometry (Å, °) *Cg*2 is the centroid of the C4–C9 benzene ring.

*D*—H⋯*A*	*D*—H	H⋯*A*	*D*⋯*A*	*D*—H⋯*A*
O3—H3*A*⋯N2^i^	0.85 (3)	2.08 (3)	2.916 (3)	170 (3)
C5—H5⋯O2^ii^	0.93	2.52	3.429 (3)	165
C2—H2*B*⋯*Cg*2^i^	0.97	2.68	3.465 (2)	138

Symmetry codes: (i) 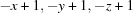; (ii) 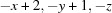.

**Fig. 1.**
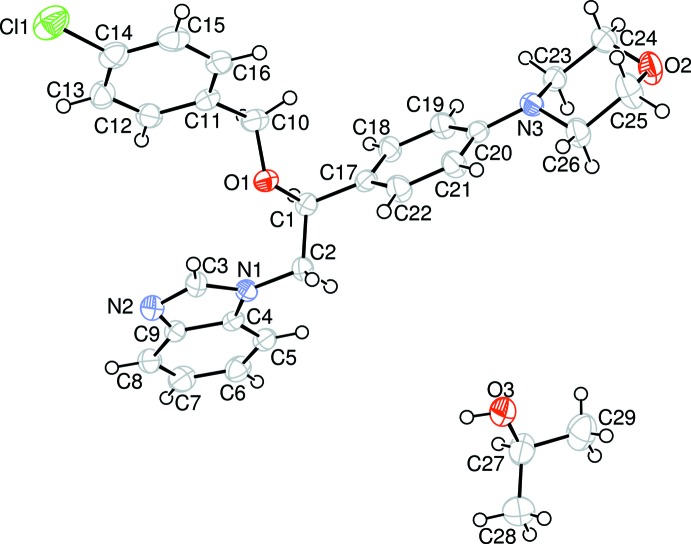
The molecular structure of the title compound with the atom-numbering scheme. Displacement ellipsoids are drawn at the 50% probability level.

**Fig. 2.**
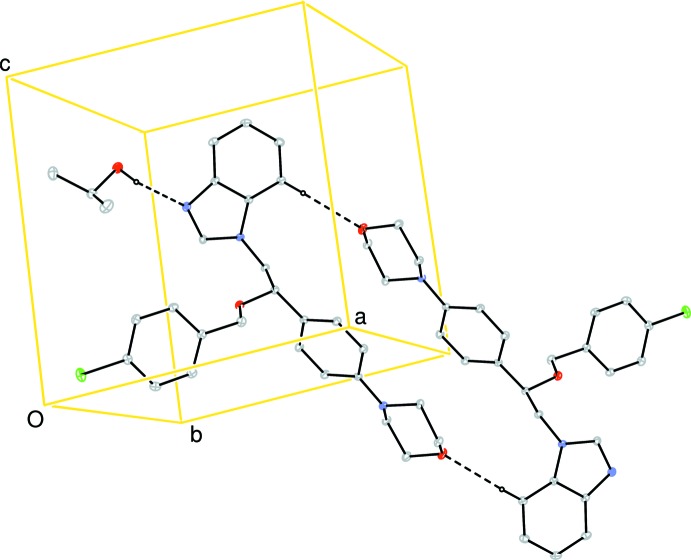
A partial packing diagram. Hydrogen bonds are shown as dashed lines. Hydrogen atoms not involved in hydrogen bonding have been omitted for clarity.

## supplementary crystallographic information

### 1. Comment

Econazole, miconazole, ketoconazole, fluconazole and itraconazole possessing imidazole or triazole ring in their structures have been known as antifungal agents and used in clinics. The crystal structures of econazole (Freer *et al.*, 1986), miconazole (Peeters *et al.*, 1979*a*), ketoconazole (Peeters *et al.*, 1979*b*), fluconazole (Caira *et al.*, 2004) and itraconazole (Peeters *et al.*, 1996) have been reported, previously. Then, similar ether structures possessing benzimidazole ring in their structures have been reported to show antibacterial activity more than antifungal ativity (Özel Güven *et al.*, 2007**a*,b*) and the crystal structures of these compounds have been reported (Özel Güven *et al.*, 2008**a*,*b*,*c*,d*). Lately, the crystal structure of a similar new compound has been reported (Özel Güven *et al.*, 2013). Now, we report herein the crystal structure of the title compound, (I), which is another benzimidazole derivative.

In the molecule of the title compound, (Fig. 1), the bond lengths and angles are generally within normal ranges. The benzimidazole [A (N1/N2/C3—C9)] ring system is approximately planar with a maximum deviation of -0.018 (2) Å for atom C6 and its mean plane is oriented with respect to the benzene [B (C11—C16)] and phenyl [C (C17—C22)] rings at dihedral angles of A/B = 4.51 (6) and A/C = 56.16 (6) °. The dihedral angle between benzene and phenyl rings is B/C = 59.11 (7)°. Atom C10 is 0.059 (2) Å away from the plane of the benzene ring and atoms C1 and N3 are 0.052 (2) and 0.084 (2) Å away from the plane of the phenyl ring. The morpholine ring D (C23—C26/O2/N3) is not planar, but adopting a chair conformation with puckering parameters (Cremer & Pople, 1975) Q_T_ = 1.044 (6)Å, φ = 33.3 (2)° and θ = 58.6 (2)°.

In the crystal structure, weak intermolecular C—H···O hydrogen bonds (Table 1) link the molecules into centrosymmetric R_2_^2^(28) dimers (Bernstein *et al.*, 1995). These dimers are further connected *via* intermolecular O—H···N hydrogen bonds to the solvent molecules (Table 1 and Fig. 2). There also exists a π···π contact between the benzene rings, Cg3—Cg3^i^, [centroid-centroid distance = 3.792 (1) Å; symmetry code: (i) 1 - x, -y, -z; Cg3 is the centroid of the ring B (C11—C16)] and two weak C—H···π interactions (Table 1), in which they may further stabilize the structure.

### 2. Experimental

The title compound, (I), was synthesized by the reaction of 2-(1*H*-benzimidazol-1-yl)-1-(4-morpholinophenyl)ethanol with aryl halide using sodium hydride. NaH (0.022 g, 0.557 mmol) was added to a solution of alcohol (0.180 g, 0.557 mmol) in DMF (4 ml) in small fractions. After stirring the mixture a few minutes, 4-chlorobenzylbromide (0.114 g, 0.557 mmol) was added. Then, the reaction mixture was stirred additional 4 h at room temperature. The reaction was stopped by adding a small amount of methyl alcohol. After evaporation of the solvent, dichloromethane was added to the reaction mixture and extracted with water. The organic phase was separated and dried with anhydrous magnesium sulfate, then evaporated to dryness. The residue was purified by column chromatography using chloroform and crystallized from isopropyl alcohol to obtain colorless crystals suitable for X-ray analysis (yield; 0.127 g, 51%).

### 3. Refinement

Atom H3A (for OH group) was located in a difference Fourier map and was freely refined. The C-bound H-atoms were positioned geometrically with C—H = 0.98, 0.93, 0.97 and 0.96 Å for methine, aromatic, methylene and methyl H, respectively, and constrained to ride on their parent atoms, with *U*_iso_(H) = k × *U*_eq_(C), where k = 1.5 for methyl H-atoms and k = 1.2 for all other H-atoms.

### Crystal data

**Table:**

C_26_H_26_ClN_3_O_2_·C_3_H_8_O	*Z* = 2
*M**_r_* = 508.04	*F*(000) = 540
Triclinic, *P*1	*D*_x_ = 1.248 Mg m^−^^3^
Hall symbol: -P 1	Mo *K*α radiation, λ = 0.71073 Å
*a* = 10.6542 (3) Å	Cell parameters from 10744 reflections
*b* = 11.5152 (4) Å	θ = 3.0–27.4°
*c* = 11.6853 (4) Å	µ = 0.18 mm^−^^1^
α = 87.010 (3)°	*T* = 294 K
β = 83.703 (3)°	Prism, colorless
γ = 71.572 (2)°	0.30 × 0.28 × 0.25 mm
*V* = 1351.66 (8) Å^3^	

### Data collection

**Table:**

Rigaku R-AXIS RAPID-S diffractometer	6139 independent reflections
Radiation source: fine-focus sealed tube	3561 reflections with *I* > 2σ(*I*)
Graphite monochromator	*R*_int_ = 0.048
ω scans	θ_max_ = 27.5°, θ_min_ = 3.0°
Absorption correction: multi-scan (*CrystalClear-SM Expert*; Rigaku, 2011)	*h* = −13→13
*T*_min_ = 0.95, *T*_max_ = 0.96	*k* = −14→14
17368 measured reflections	*l* = −14→15

### Refinement

**Table:**

Refinement on *F*^2^	Secondary atom site location: difference Fourier map
Least-squares matrix: full	Hydrogen site location: inferred from neighbouring sites
*R*[*F*^2^ > 2σ(*F*^2^)] = 0.055	H atoms treated by a mixture of independent and constrained refinement
*wR*(*F*^2^) = 0.186	*w* = 1/[σ^2^(*F*_o_^2^) + (0.093*P*)^2^] where *P* = (*F*_o_^2^ + 2*F*_c_^2^)/3
*S* = 1.11	(Δ/σ)_max_ < 0.001
6139 reflections	Δρ_max_ = 0.50 e Å^−^^3^
332 parameters	Δρ_min_ = −0.53 e Å^−^^3^
1 restraint	Extinction correction: *SHELXL97* (Sheldrick, 2008), Fc^*^=kFc[1+0.001xFc^2^λ^3^/sin(2θ)]^-1/4^
Primary atom site location: structure-invariant direct methods	Extinction coefficient: 0.010 (3)

### Special details

**Table:**

Geometry. All esds (except the esd in the dihedral angle between two l.s. planes) are estimated using the full covariance matrix. The cell esds are taken into account individually in the estimation of esds in distances, angles and torsion angles; correlations between esds in cell parameters are only used when they are defined by crystal symmetry. An approximate (isotropic) treatment of cell esds is used for estimating esds involving l.s. planes.
Refinement. Refinement of F^2^ against ALL reflections. The weighted R-factor wR and goodness of fit S are based on F^2^, conventional R-factors R are based on F, with F set to zero for negative F^2^. The threshold expression of F^2^ > 2sigma(F^2^) is used only for calculating R-factors(gt) etc. and is not relevant to the choice of reflections for refinement. R-factors based on F^2^ are statistically about twice as large as those based on F, and R- factors based on ALL data will be even larger.

### Fractional atomic coordinates and isotropic or equivalent isotropic displacement parameters (Å^2^)

**Table:**

	*x*	*y*	*z*	*U*_iso_*/*U*_eq_	
Cl1	0.12222 (6)	0.01131 (6)	0.06222 (7)	0.0564 (3)	
O1	0.55011 (15)	0.28048 (12)	0.19797 (12)	0.0332 (4)	
O2	1.05293 (17)	0.58050 (15)	−0.36418 (15)	0.0478 (5)	
O3	0.73878 (19)	0.85398 (16)	0.32068 (16)	0.0496 (5)	
H3A	0.699 (3)	0.816 (3)	0.368 (2)	0.086 (12)*	
N1	0.55093 (18)	0.35870 (15)	0.42231 (14)	0.0296 (4)	
N2	0.41749 (19)	0.27808 (17)	0.53940 (16)	0.0364 (5)	
N3	0.90888 (18)	0.48375 (15)	−0.18415 (15)	0.0313 (4)	
C1	0.6596 (2)	0.31728 (19)	0.22470 (18)	0.0308 (5)	
H1	0.7254	0.2463	0.2565	0.037*	
C2	0.6021 (2)	0.41068 (19)	0.31857 (18)	0.0317 (5)	
H2A	0.6705	0.4436	0.3373	0.038*	
H2B	0.5308	0.4777	0.2902	0.038*	
C3	0.4401 (2)	0.32288 (19)	0.4366 (2)	0.0337 (5)	
H3	0.3851	0.3296	0.3785	0.040*	
C4	0.6061 (2)	0.33516 (18)	0.52643 (18)	0.0299 (5)	
C5	0.7173 (2)	0.35401 (19)	0.56430 (19)	0.0357 (5)	
H5	0.7736	0.3863	0.5157	0.043*	
C6	0.7403 (3)	0.3225 (2)	0.6777 (2)	0.0433 (6)	
H6	0.8127	0.3352	0.7066	0.052*	
C7	0.6563 (3)	0.2716 (2)	0.7504 (2)	0.0462 (7)	
H7	0.6750	0.2508	0.8260	0.055*	
C8	0.5472 (3)	0.2517 (2)	0.7123 (2)	0.0413 (6)	
H8	0.4926	0.2174	0.7608	0.050*	
C9	0.5211 (2)	0.28481 (18)	0.59858 (19)	0.0328 (5)	
C10	0.5907 (3)	0.1737 (2)	0.1282 (2)	0.0419 (6)	
H10A	0.6551	0.1073	0.1652	0.050*	
H10B	0.6318	0.1911	0.0538	0.050*	
C11	0.4708 (2)	0.13819 (19)	0.1133 (2)	0.0343 (5)	
C12	0.4139 (2)	0.0811 (2)	0.2031 (2)	0.0376 (6)	
H12	0.4498	0.0671	0.2735	0.045*	
C13	0.3050 (2)	0.0453 (2)	0.1886 (2)	0.0398 (6)	
H13	0.2670	0.0078	0.2490	0.048*	
C14	0.2527 (2)	0.0656 (2)	0.0829 (2)	0.0389 (6)	
C15	0.3053 (3)	0.1241 (2)	−0.0070 (2)	0.0406 (6)	
H15	0.2691	0.1383	−0.0772	0.049*	
C16	0.4130 (3)	0.1608 (2)	0.0104 (2)	0.0389 (6)	
H16	0.4480	0.2020	−0.0489	0.047*	
C17	0.7259 (2)	0.36472 (18)	0.12031 (18)	0.0301 (5)	
C18	0.8556 (2)	0.30403 (19)	0.07960 (19)	0.0349 (5)	
H18	0.9027	0.2344	0.1190	0.042*	
C19	0.9176 (2)	0.3435 (2)	−0.0178 (2)	0.0372 (6)	
H19	1.0055	0.3004	−0.0419	0.045*	
C20	0.8508 (2)	0.44694 (18)	−0.08082 (18)	0.0297 (5)	
C21	0.7187 (2)	0.5080 (2)	−0.0392 (2)	0.0368 (6)	
H21	0.6704	0.5769	−0.0789	0.044*	
C22	0.6590 (2)	0.4684 (2)	0.0586 (2)	0.0376 (6)	
H22	0.5718	0.5119	0.0843	0.045*	
C23	1.0515 (2)	0.4257 (2)	−0.2155 (2)	0.0358 (6)	
H23A	1.0739	0.3379	−0.2027	0.043*	
H23B	1.1021	0.4564	−0.1674	0.043*	
C24	1.0869 (3)	0.4525 (2)	−0.3397 (2)	0.0434 (6)	
H24A	1.1817	0.4149	−0.3589	0.052*	
H24B	1.0408	0.4165	−0.3876	0.052*	
C25	0.9129 (3)	0.6344 (2)	−0.3385 (2)	0.0512 (7)	
H25A	0.8669	0.5993	−0.3874	0.061*	
H25B	0.8889	0.7216	−0.3555	0.061*	
C26	0.8684 (2)	0.6145 (2)	−0.2142 (2)	0.0405 (6)	
H26A	0.9071	0.6566	−0.1652	0.049*	
H26B	0.7724	0.6485	−0.2012	0.049*	
C27	0.8136 (3)	0.8985 (2)	0.3906 (2)	0.0507 (7)	
H27	0.8611	0.8314	0.4402	0.061*	
C28	0.7247 (4)	1.0008 (3)	0.4653 (3)	0.0780 (11)	
H28A	0.6592	0.9727	0.5113	0.117*	
H28B	0.6810	1.0687	0.4175	0.117*	
H28C	0.7770	1.0261	0.5148	0.117*	
C29	0.9145 (3)	0.9384 (3)	0.3103 (3)	0.0661 (9)	
H29A	0.9703	0.8700	0.2660	0.099*	
H29B	0.9681	0.9684	0.3546	0.099*	
H29C	0.8690	1.0023	0.2594	0.099*	

### Atomic displacement parameters (Å^2^)

**Table:**

	*U*^11^	*U*^22^	*U*^33^	*U*^12^	*U*^13^	*U*^23^
Cl1	0.0380 (4)	0.0487 (4)	0.0835 (6)	−0.0127 (3)	−0.0057 (4)	−0.0166 (4)
O1	0.0351 (9)	0.0301 (8)	0.0351 (9)	−0.0117 (7)	0.0014 (7)	−0.0076 (6)
O2	0.0428 (11)	0.0463 (10)	0.0497 (11)	−0.0139 (8)	0.0105 (8)	0.0080 (8)
O3	0.0585 (13)	0.0505 (11)	0.0468 (11)	−0.0286 (9)	0.0008 (9)	−0.0029 (9)
N1	0.0369 (11)	0.0313 (9)	0.0229 (9)	−0.0140 (8)	−0.0027 (8)	−0.0003 (7)
N2	0.0369 (12)	0.0379 (10)	0.0370 (11)	−0.0162 (9)	0.0005 (9)	−0.0032 (8)
N3	0.0332 (11)	0.0306 (9)	0.0286 (10)	−0.0086 (8)	0.0002 (8)	−0.0013 (8)
C1	0.0350 (13)	0.0272 (11)	0.0310 (12)	−0.0113 (9)	−0.0026 (10)	0.0000 (9)
C2	0.0381 (13)	0.0316 (11)	0.0278 (11)	−0.0151 (10)	−0.0018 (10)	0.0022 (9)
C3	0.0336 (13)	0.0366 (12)	0.0348 (12)	−0.0174 (10)	−0.0008 (10)	−0.0003 (10)
C4	0.0360 (13)	0.0231 (10)	0.0312 (12)	−0.0100 (9)	−0.0019 (10)	−0.0048 (9)
C5	0.0428 (14)	0.0311 (12)	0.0350 (13)	−0.0139 (10)	−0.0036 (11)	−0.0024 (10)
C6	0.0534 (16)	0.0402 (13)	0.0407 (14)	−0.0187 (12)	−0.0123 (12)	0.0009 (11)
C7	0.0681 (19)	0.0421 (14)	0.0314 (13)	−0.0194 (13)	−0.0130 (12)	0.0045 (11)
C8	0.0579 (17)	0.0337 (12)	0.0325 (13)	−0.0168 (11)	0.0015 (12)	0.0021 (10)
C9	0.0423 (14)	0.0252 (10)	0.0308 (12)	−0.0125 (10)	0.0035 (10)	−0.0018 (9)
C10	0.0459 (15)	0.0383 (13)	0.0435 (14)	−0.0165 (11)	0.0041 (12)	−0.0146 (11)
C11	0.0410 (14)	0.0254 (11)	0.0375 (13)	−0.0122 (9)	0.0005 (11)	−0.0078 (9)
C12	0.0474 (15)	0.0323 (12)	0.0316 (12)	−0.0114 (10)	−0.0006 (11)	−0.0015 (10)
C13	0.0426 (15)	0.0296 (12)	0.0463 (15)	−0.0123 (10)	0.0022 (12)	0.0001 (10)
C14	0.0363 (14)	0.0279 (11)	0.0492 (15)	−0.0051 (10)	−0.0028 (11)	−0.0071 (10)
C15	0.0503 (16)	0.0302 (12)	0.0367 (14)	−0.0041 (11)	−0.0081 (12)	−0.0045 (10)
C16	0.0520 (16)	0.0299 (12)	0.0349 (13)	−0.0139 (11)	0.0010 (11)	−0.0056 (10)
C17	0.0379 (13)	0.0252 (10)	0.0292 (11)	−0.0125 (9)	−0.0045 (10)	−0.0007 (9)
C18	0.0381 (14)	0.0286 (11)	0.0342 (12)	−0.0068 (10)	−0.0013 (10)	0.0052 (9)
C19	0.0308 (13)	0.0344 (12)	0.0405 (13)	−0.0033 (10)	0.0010 (10)	−0.0001 (10)
C20	0.0344 (13)	0.0270 (10)	0.0298 (11)	−0.0116 (9)	−0.0048 (10)	−0.0022 (9)
C21	0.0377 (14)	0.0305 (11)	0.0348 (13)	−0.0028 (10)	0.0016 (10)	0.0044 (10)
C22	0.0341 (13)	0.0327 (12)	0.0392 (13)	−0.0033 (10)	0.0037 (11)	0.0014 (10)
C23	0.0334 (13)	0.0314 (11)	0.0410 (13)	−0.0102 (10)	0.0049 (11)	−0.0033 (10)
C24	0.0434 (15)	0.0435 (14)	0.0414 (14)	−0.0147 (11)	0.0095 (12)	−0.0052 (11)
C25	0.0427 (16)	0.0558 (16)	0.0449 (15)	−0.0063 (12)	0.0032 (12)	0.0159 (13)
C26	0.0395 (14)	0.0363 (13)	0.0390 (14)	−0.0063 (10)	0.0042 (11)	0.0071 (10)
C27	0.0587 (18)	0.0490 (15)	0.0509 (16)	−0.0259 (13)	−0.0093 (14)	0.0071 (13)
C28	0.112 (3)	0.073 (2)	0.060 (2)	−0.050 (2)	0.0162 (19)	−0.0216 (17)
C29	0.0525 (19)	0.0605 (18)	0.089 (2)	−0.0247 (15)	−0.0039 (17)	0.0047 (16)

### Geometric parameters (Å, º)

**Table:**

Cl1—C14	1.738 (3)	C14—C15	1.381 (3)
O1—C1	1.430 (3)	C14—C13	1.389 (4)
O1—C10	1.433 (2)	C15—C16	1.380 (3)
O2—C24	1.424 (3)	C15—H15	0.9300
O2—C25	1.427 (3)	C16—C11	1.386 (3)
O3—C27	1.418 (3)	C16—H16	0.9300
O3—H3A	0.847 (18)	C17—C1	1.507 (3)
N1—C2	1.453 (3)	C17—C18	1.379 (3)
N1—C3	1.360 (3)	C17—C22	1.392 (3)
N1—C4	1.386 (3)	C18—H18	0.9300
N2—C3	1.310 (3)	C19—C18	1.382 (3)
N2—C9	1.389 (3)	C19—C20	1.398 (3)
N3—C20	1.403 (3)	C19—H19	0.9300
N3—C23	1.466 (3)	C20—C21	1.404 (3)
N3—C26	1.464 (3)	C21—H21	0.9300
C1—C2	1.515 (3)	C22—C21	1.375 (3)
C1—H1	0.9800	C22—H22	0.9300
C2—H2A	0.9700	C23—C24	1.500 (3)
C2—H2B	0.9700	C23—H23A	0.9700
C3—H3	0.9300	C23—H23B	0.9700
C4—C5	1.391 (3)	C24—H24A	0.9700
C4—C9	1.407 (3)	C24—H24B	0.9700
C5—C6	1.383 (3)	C25—H25A	0.9700
C5—H5	0.9300	C25—H25B	0.9700
C6—H6	0.9300	C26—C25	1.507 (3)
C7—C6	1.406 (3)	C26—H26A	0.9700
C7—H7	0.9300	C26—H26B	0.9700
C8—C7	1.376 (4)	C27—C28	1.506 (4)
C8—H8	0.9300	C27—C29	1.514 (4)
C9—C8	1.397 (3)	C27—H27	0.9800
C10—H10A	0.9700	C28—H28A	0.9600
C10—H10B	0.9700	C28—H28B	0.9600
C11—C10	1.489 (3)	C28—H28C	0.9600
C12—C11	1.394 (3)	C29—H29A	0.9600
C12—C13	1.378 (3)	C29—H29B	0.9600
C12—H12	0.9300	C29—H29C	0.9600
C13—H13	0.9300		
			
C1—O1—C10	112.78 (16)	C11—C16—H16	119.0
C24—O2—C25	108.72 (18)	C15—C16—C11	122.1 (2)
C27—O3—H3A	103 (2)	C15—C16—H16	119.0
C3—N1—C2	127.01 (18)	C18—C17—C1	120.85 (19)
C3—N1—C4	106.32 (17)	C18—C17—C22	117.0 (2)
C4—N1—C2	126.68 (18)	C22—C17—C1	122.07 (19)
C3—N2—C9	104.40 (19)	C17—C18—C19	122.1 (2)
C20—N3—C23	118.45 (17)	C17—C18—H18	119.0
C20—N3—C26	117.93 (16)	C19—C18—H18	119.0
C26—N3—C23	111.22 (17)	C18—C19—C20	121.2 (2)
O1—C1—C17	112.39 (17)	C18—C19—H19	119.4
O1—C1—C2	104.93 (17)	C20—C19—H19	119.4
O1—C1—H1	108.5	N3—C20—C21	121.36 (19)
C2—C1—H1	108.5	C19—C20—N3	122.12 (19)
C17—C1—C2	113.76 (17)	C19—C20—C21	116.4 (2)
C17—C1—H1	108.5	C20—C21—H21	119.2
N1—C2—C1	112.38 (17)	C22—C21—C20	121.6 (2)
N1—C2—H2A	109.1	C22—C21—H21	119.2
N1—C2—H2B	109.1	C17—C22—H22	119.2
C1—C2—H2A	109.1	C21—C22—C17	121.6 (2)
C1—C2—H2B	109.1	C21—C22—H22	119.2
H2A—C2—H2B	107.9	N3—C23—C24	110.09 (19)
N1—C3—H3	122.9	N3—C23—H23A	109.6
N2—C3—N1	114.2 (2)	N3—C23—H23B	109.6
N2—C3—H3	122.9	C24—C23—H23A	109.6
N1—C4—C5	132.8 (2)	C24—C23—H23B	109.6
N1—C4—C9	105.00 (19)	H23A—C23—H23B	108.2
C5—C4—C9	122.2 (2)	O2—C24—C23	112.08 (18)
C4—C5—H5	121.6	O2—C24—H24A	109.2
C6—C5—C4	116.9 (2)	O2—C24—H24B	109.2
C6—C5—H5	121.6	C23—C24—H24A	109.2
C5—C6—C7	121.4 (2)	C23—C24—H24B	109.2
C5—C6—H6	119.3	H24A—C24—H24B	107.9
C7—C6—H6	119.3	O2—C25—C26	112.0 (2)
C6—C7—H7	119.2	O2—C25—H25A	109.2
C8—C7—C6	121.7 (2)	O2—C25—H25B	109.2
C8—C7—H7	119.2	C26—C25—H25A	109.2
C7—C8—C9	117.8 (2)	C26—C25—H25B	109.2
C7—C8—H8	121.1	H25A—C25—H25B	107.9
C9—C8—H8	121.1	N3—C26—C25	110.64 (19)
N2—C9—C4	110.1 (2)	N3—C26—H26A	109.5
N2—C9—C8	129.9 (2)	N3—C26—H26B	109.5
C8—C9—C4	120.0 (2)	C25—C26—H26A	109.5
O1—C10—C11	108.21 (18)	C25—C26—H26B	109.5
O1—C10—H10A	110.1	H26A—C26—H26B	108.1
O1—C10—H10B	110.1	O3—C27—C28	111.1 (2)
C11—C10—H10A	110.1	O3—C27—C29	106.9 (2)
C11—C10—H10B	110.1	O3—C27—H27	108.8
H10A—C10—H10B	108.4	C28—C27—C29	112.4 (2)
C12—C11—C10	120.5 (2)	C28—C27—H27	108.8
C16—C11—C10	121.2 (2)	C29—C27—H27	108.8
C16—C11—C12	118.4 (2)	C27—C28—H28A	109.5
C11—C12—H12	119.7	C27—C28—H28B	109.5
C13—C12—C11	120.6 (2)	C27—C28—H28C	109.5
C13—C12—H12	119.7	H28A—C28—H28B	109.5
C12—C13—C14	119.4 (2)	H28A—C28—H28C	109.5
C12—C13—H13	120.3	H28B—C28—H28C	109.5
C14—C13—H13	120.3	C27—C29—H29A	109.5
C13—C14—Cl1	119.17 (19)	C27—C29—H29B	109.5
C15—C14—Cl1	119.5 (2)	C27—C29—H29C	109.5
C15—C14—C13	121.3 (2)	H29A—C29—H29B	109.5
C14—C15—H15	120.9	H29A—C29—H29C	109.5
C16—C15—C14	118.2 (2)	H29B—C29—H29C	109.5
C16—C15—H15	120.9		
			
C10—O1—C1—C17	68.3 (2)	C8—C7—C6—C5	−0.5 (4)
C10—O1—C1—C2	−167.60 (17)	C9—C8—C7—C6	−0.5 (4)
C1—O1—C10—C11	175.73 (18)	N2—C9—C8—C7	−178.2 (2)
C25—O2—C24—C23	−60.4 (3)	C4—C9—C8—C7	0.8 (3)
C24—O2—C25—C26	59.3 (3)	C12—C11—C10—O1	−75.2 (3)
C3—N1—C2—C1	−70.9 (3)	C16—C11—C10—O1	104.8 (2)
C4—N1—C2—C1	108.7 (2)	C13—C12—C11—C16	1.7 (3)
C2—N1—C3—N2	179.53 (18)	C13—C12—C11—C10	−178.3 (2)
C4—N1—C3—N2	−0.2 (2)	C11—C12—C13—C14	0.5 (3)
C2—N1—C4—C5	1.5 (4)	Cl1—C14—C13—C12	175.85 (17)
C2—N1—C4—C9	−179.65 (18)	C15—C14—C13—C12	−1.8 (3)
C3—N1—C4—C5	−178.8 (2)	Cl1—C14—C15—C16	−176.86 (17)
C3—N1—C4—C9	0.0 (2)	C13—C14—C15—C16	0.8 (3)
C9—N2—C3—N1	0.2 (2)	C14—C15—C16—C11	1.5 (3)
C3—N2—C9—C4	−0.2 (2)	C15—C16—C11—C10	177.2 (2)
C3—N2—C9—C8	178.9 (2)	C15—C16—C11—C12	−2.8 (3)
C23—N3—C20—C19	−11.4 (3)	C18—C17—C1—O1	−115.5 (2)
C23—N3—C20—C21	172.8 (2)	C18—C17—C1—C2	125.5 (2)
C26—N3—C20—C19	−150.3 (2)	C22—C17—C1—O1	62.4 (3)
C26—N3—C20—C21	33.9 (3)	C22—C17—C1—C2	−56.7 (3)
C20—N3—C23—C24	165.53 (18)	C1—C17—C18—C19	178.1 (2)
C26—N3—C23—C24	−53.0 (2)	C22—C17—C18—C19	0.1 (3)
C20—N3—C26—C25	−166.0 (2)	C1—C17—C22—C21	−177.3 (2)
C23—N3—C26—C25	52.4 (3)	C18—C17—C22—C21	0.7 (3)
O1—C1—C2—N1	62.6 (2)	C20—C19—C18—C17	−0.7 (4)
C17—C1—C2—N1	−174.18 (18)	C18—C19—C20—N3	−175.6 (2)
N1—C4—C5—C6	177.8 (2)	C18—C19—C20—C21	0.3 (3)
C9—C4—C5—C6	−0.9 (3)	N3—C20—C21—C22	176.5 (2)
N1—C4—C9—N2	0.1 (2)	C17—C22—C21—C20	−1.0 (4)
N1—C4—C9—C8	−179.09 (19)	C19—C20—C21—C22	0.5 (3)
C5—C4—C9—N2	179.06 (18)	N3—C23—C24—O2	57.8 (3)
C5—C4—C9—C8	−0.1 (3)	N3—C26—C25—O2	−56.1 (3)
C4—C5—C6—C7	1.2 (3)		

### Hydrogen-bond geometry (Å, º)

Cg2 is the centroid of the C4–C9 benzene ring.

**Table:**

*D*—H···*A*	*D*—H	H···*A*	*D*···*A*	*D*—H···*A*
O3—H3*A*···N2^i^	0.85 (3)	2.08 (3)	2.916 (3)	170 (3)
C5—H5···O2^ii^	0.93	2.52	3.429 (3)	165
C2—H2*B*···*Cg*2^i^	0.97	2.68	3.465 (2)	138

Symmetry codes: (i) −*x*+1, −*y*+1, −*z*+1; (ii) −*x*+2, −*y*+1, −*z*.
